# Case of Popliteal Aneurism

**Published:** 1807-04

**Authors:** 


### *To the Editors of the Medical and Physical Journal.*

Gentlemen,

I Must confess, it requires no common fortitude for a man hazarding his opinion in your Journal. The spirit of controversy, and the indulgence of illiberal suggestions, have, I am sorry to say, become so prevalent of late, that any person who communicates information relative to our uncertain profession, must be upon his guard; for if he deviates in a solitary instance from grammatical precision, he lays himself open to criticism. I abhor personality, because I am averse to enter the controversial list; but I must be blind indeed, if I did not perceive in the writings of many of your correspondents, a spirit of opposition unworthy of the profession. It is not by illiberality that we are to arrive at the truth; nor will stabbing a neighbour's reputation, produce improvement of our art. No, it is by observing well and accurately every untoward appearance, pearance, and every deviation that disease takes from Nature's common path; that can ensure success in the advancement of science, unaccompanied by rudeness, or scurrility.

Situated at a Naval Hospital, it necessarily must fall to my lot to witness the professional skill of Naval Surgeons; and I beg leave to say, I know no set of men, taken as a body, who deserve more of their country. They sacrifice every pleasure, and every domestic comfort, for the welfare of the state. It is true, that their constant attendance on a London Hospital must be dispensed with, owing to their attendance in the service of their country, consequently they cannot be masters of the most recent improvements of Surgery. But I will venture to tell the profession a truth, which is, that the practice of Surgery is *capricious.* I am acquainted with many eminent Naval Surgeons, who cannot, from diffidence or want of opportunity, blaze forth their names in the periodical publications of the day, who, nevertheless, bestow great comfort on their patients, and perform cures while labouring under every disadvantage, which would do honour to men surrounded with all the benefits that books and instruments can bestow. Many men are clear in their ideas of practice, at the same time perplexed in their way of reasoning. It should be a maxim held sacred, to record the unsuccessful cases with the more successful ones, and narrate the former as faithfully as the latter. By strictly observing this, I am persuaded we should practice with much more certainty a profession so closely allied to the interest of mankind. Gentlemen, who bestow information on their brethren, instead of having their feelings injured, should be supported; they can have no view but the benefit and welfare of society; and if the garb in which such information is dressed, does not at all times boast of classical purity, or logical accuracy, yet it may prove ultimately of much value. The plain unvarnished Tale is best.

With the foregoing observations on Illiberality, I transmit two Cases; one of Popliteal Aneurism, and the other a Case of Mollities Ossium. As they appear to me to be interesting, I should feel myself obliged and gratified if any of your Correspondents would offer their Remarks thereon with *candour.*

### CASE I.

James Burn, able seaman, was admitted into the Royal Hospital, Deal, 1805, on account of a popliteal aneurism. The pulsation of the aneurismal tumour was very strong, and and upon applying the fingers, accompanied by a moderate pressure, the blood could be very easily felt flowing or trickling, (a term which, perhaps, will convey a better idea of the subject) into the sac.

The only account the man could give concerning it, was, that in the act of heaving up the anchor, his foot slipped, and the moment that it did so, he felt something snap, and was instantly seized with a most violent pain, which was so severe, that it rendered him totally unable to go on with his duty, and obliged him to retire. This took place about 5 or 6 weeks previous to his admission into the Hospital. Upon his entrance, his temperament was such, as seemed highly predisposed to the disease, it being sanguineous. The patient was ordered to his bed, put upon a low diet, and at the same time, in order to reduce the constitution, and allay the impetus of the circulation, antimonials with opiates were prescribed. The tumor appeared stationary for about a fortnight; but at the expiration of that time, it began very visibly to encrease. Œdema of the leg came on, which extended itself to the foot, accompanied with numbness. The muscles of the thigh began to diminish, and the patient's constitution to be very much reduced. As there could be no doubt about the nature of the disease, the operation was resolved upon, and accordingly performed on Monday the 18th of March.—Describing the operation may appear superfluous; but at the same time may prove satisfactory.

OPERATION.

An incision, about two inches and a half in length, was made between the inner edge of the Sartorius and long-head of the triceps; the cellular membrane was next divided, and upon its division, the theca or sheath which encloses the femoral artery, nerve and vein, was brought into sight; the pulsation of the former being very evident. The sheath was very gently divided, which of course prevented the vessels from having so close an alliance. An eye probe, having one end of a ligature passed through its opening, at the other end of which, a moderate size curved needle was placed, it was then forced through the condensed cellular membrane which binds down the artery; the latter was then separated from the vein and nerve, and another ligature was introduced under the vessel; great care being taken, not to strip the artery more than was necessary of its surrounding cellular structure. The upper one was then tied, and as low down as the detachment of the the artery would allow, the lower ligature was tied. The needle was then run through the inferior part of the artery, about half an inch below the first ligature. Close to the perforation made by the needle, was the femoral artery divided; that part which was situated between the first ligature and the perforation was then brought over the artery and firmly secured. The divided integuments were brought together by means of the adhesive plaster; and after the application of the roller, the patient was put to bed.

The patient complained of great numbness, and said that his leg and foot were as cold as ice, but more particularly the latter. These two last symptoms vanished gradually, and at the end of three days had entirely subsided. The bandage and superficial dressing were removed on the fourth day after the operation, but the straps of sticking plaster were suffered to remain. The man appeared much better; talked with confidence about his recovery; and indeed every circumstance augured favourably. On the sixth day, the straps were removed, and the wound had very much to our satisfaction healed by the first intention; there being but a very trifling discharge, and that came only from the openings where the ligatures were brought out. On the twelfth day, the lower ligature came away, and the upper one on the fourteenth. External stimulants were employed about the tumour, with a view of hastening absorption; but an eschar of the size of half a crown, appeared at its most depending part. This eschar sloughed away in a few days, and gave exit to a large quantity of thick coagulated blood, that smelt extremely offensive. Warm milk and water was injected by this opening, and brought away a considerable quantity of this thick coagulum.

The injection having been thrown into the sac in the morning about eight o'clock; the same evening, while he was returning from the close stool, a hæmorrhage took place, and in the course of half a minute he lost at least four pounds of blood. The Assistant Surgeon happened to be in the Ward, and a tourniquet at hand was immediately applied. The compression that was required to be made, in order to stop the hæmorrhage, was very great, but ineffectual to a certain degree; and he lost some quantity after the most severe pressure was applied, that possibly could be made. As it would have been the height of madness and folly to search for vessels in such a mass of corruption, the poor fellow being in a complete state of deliquium deliquium animi, from so sudden a loss, that a quick determination was necessary, as to the manner of proceeding. The only alternative, therefore, was amputation, which was immediately performed. He lost very little blood during amputation, although the arteries that were taken up, amounted to twelve. He appeared perfectly easy and composed after the operation, which must be ascribed in great measure to inanition. He remained in this state from the moment that he was placed in bed, until his dissolution, which took place about twelve o'clock the following morning, April 4th. Nothing particular was to be met with in the dissection of the limb. The aneurismal sac was found situated between the tendons of the muscles, which form the inner and outer hamstring, and the heads of the gastrocnemii muscles. The profunda was found to be much larger than the femoral artery, nearly half as large again.

REMARKS.

I shall take the liberty of suggesting my ideas relative to this unfortunate case. I hope it will appear that every thing was done which human wisdom could devise. The operation was undertaken with the most flattering prospect of success; in short, every circumstance conspired towards our forming a favourable prognosis. Perhaps it may appear to many, that the operation was deferred too long. It may have been so, but the only reason for so doing was, that the profunda and its branches should have time to dilate, in order that the circulation might be carried on with greater facility after the obliteration of the femoral artery. This circumstance must always take place in a greater or less degree, depending in a great measure on the magnitude of the aneurismal tumour. When the dilatation has taken place to a considerable degree, the blood of course cannot find an easy passage into those arteries situated below, consequently a portion of the blood must take a retrograde motion, and the collateral vessels that are given off from the main artery must increase considerably in diameter. The next point to be ascertained is, from what arteries proceeded the fatal hæmorrhage? And I think another question not undeserving attention, what was the proximate cause of the rupture? It is feasible to suppose, that the anastamosing vessels situate about the joints, were the vessels which were concerned in the hæmorrhage, and that the rupture must have been occasioned in consequence of erosion caused by the acrimony of the coagulated blood situated in the sac.

CASE

### CASE II.

Mr. Duncan Stuart, assistant surgeon of the Royal Navy, was admitted into the Hospital, labouring under great prostration of strength, accompanied with paralysis of the lower extremities. The bladder had lost its contractile power, so as to oblige us to use the catheter night and morning. The urine was turbid and high coloured, and appeared as if brick-dust was mixed with it; mucus and small particles of calcareous matter were found frequently deposited at the bottom of the urinal. He complained of dreadful and incessant pain in his back, which was evidently increased on his removing from a horizontal position to an erect one. There was great wasting of the whole of the muscles of the body, but more particularly those of the lower extremities. He had little or no appetite, and great quantities of wine and porter were taken, accompanied with tonic medicines, chiefly chalybeates. Hectic fever and sleepless nights supervened; his debility increased, and he sunk gradually into the arms of death.

### DISSECTION.

On opening the cavity of the abdomen, the appearances that presented themselves were the following. Two of the lumbar vertebræ were found decayed, the compact substance which originally formed the bodies of these bones could in this case be easily and completely divided with a scalpel; they were so soft that they had not preserved their situation, and in consequence had compressed the spinal marrow. In the bladder were found small particles of calculi, so brittle as to be easily crumbled between the finger and thumb; its internal surface was also found much inflamed. In the pelvis of each kidney was found the same earthy matter as was met with in the bladder, accompanied with a glary mucal discharge. Both these organs were very vascular. Nor were the lumbar vertebræ the only bones which partook of disease, the ribs were so soft that the uniformity of the chest was not preserved, and the cartilages pressed inwards upon the viscera. Small tumours had formed near to the angles of the ribs, which on being opened, a glutinous substance escaped. The lungs and heart were healthy, but a considerable quantity of water was found in the pericardium.

### REMARKS.

Seldom, I believe, have the ravages of this disease, Mollities Ossium, made greater progress or havoc than in the above-mentioned case, or proceeded to such great lengths, lengths, without terminating vitality much sooner. The paralysis of the lower extremities, and the inactivity of the contractile power of the bladder, are easily accounted for, in consequence of compression of the spinal marrow, caused by the carious state of the lumbar vertebræ. The particles of earthy matter, which were voided prior to dissolution, and the same substance being found lodged through the whole course of the urinary canal, denotes evidently that the lymphatics were actively employed in removing the animal earth which produces compactness of bone, and that the kidneys were the channels through which this important substance, so necessary for its organization and structure, was carried out of the system.

I am, &c.

*Royal Naval Hospital, Deal, March* 15, 1807.

